# Translational reprogramming under heat stress: a plant’s perspective

**DOI:** 10.1098/rsos.250132

**Published:** 2025-07-16

**Authors:** Moray Smith, Aminin Taqrir Akramin, Martin Balcerowicz

**Affiliations:** ^1^ Division of Plant Sciences, University of Dundee, Dundee, UK

**Keywords:** translation, translation factors, protein synthesis, heat stress, temperature sensing

## Abstract

Plants experience dynamic and sometimes extreme fluctuations in temperature on hourly, daily and seasonal scales, which are becoming increasingly challenging as climate change progresses. To maximize fitness and chances of survival, plants continuously adjust their growth, development and physiology to their temperature environment. Changes in protein synthesis are central to these acclimatization processes, enabling rapid and precise modulation of cellular functions. In this review, we discuss the molecular mechanisms driving heat-induced translational reprogramming, integrating insights from animal and yeast systems with current knowledge and emerging hypotheses in plants. We revisit the core stages of translation—initiation, elongation and termination—and the roles of associated translation factors while also exploring emerging areas of interest, including biomolecular condensates, RNA modifications and *cis*-regulatory elements. Finally, we consider how a deeper understanding of translational control could be harnessed to enhance crop resilience in the face of climate change.

## 1. Introduction

Organisms constantly interact with their environment and face a wide range of biotic and abiotic stresses that require them to adjust their growth and physiology to maintain fitness and ensure survival. Such adjustments are brought about by changes in gene expression, which not only involve widespread transcriptional reprogramming but also alterations at the level of translation. An increase in ambient temperature triggers global changes in translation efficiency in a variety of organisms, including bacteria [1,2], yeast [3], animals [4–6] and plants [7–10]. A sudden rise in intracellular temperature experienced during heat shock often results in a global shutdown of translation while selectively enhancing the synthesis of stress-related proteins such as heat shock proteins (HSPs) [11,12]. These changes are vital to prevent the accumulation of misfolded or otherwise damaged proteins that can exert proteotoxic effects and threaten survival. Efficient recovery of translation once the stress has subsided is, however, equally important to maintain fitness.

The mechanisms underlying high temperature-induced changes in translation—both transcriptome-wide and for specific transcripts—are incompletely understood. Several regulatory processes have been identified in prokaryotic systems [13–15], but in eukaryotes, particularly plants, these mechanisms are just beginning to emerge. In this review, we provide an overview of the translation process in eukaryotes and examine the current understanding of how translation is regulated in response to elevated temperature, contrasting insight into plant-specific translational mechanisms with processes known from yeast and animal systems. We revisit the involvement of translation factors in the processes of cap-dependent and cap-independent translation and explore emerging areas of interest, including the role of biomolecular condensates, RNA modifications and *cis*-regulatory RNA elements. We conclude by discussing the potential of translational regulation as a promising but largely unexplored avenue for improving crop resilience in a warming climate.

## 2. Eukaryotic translation under non-stress conditions

Translation is a fundamental cellular process that synthesizes proteins and involves three main phases: initiation, elongation and termination. It occurs in the cytoplasm, where the major components—messenger RNAs (mRNAs), ribosomes, transfer RNAs (tRNAs) and translation factors—coordinate to convert the nucleotide sequence of mRNA into a functional protein. The initiation process begins with the assembly of the pre-initiation complex (PIC), which scans the mRNA to locate the start codon within a favourable sequence context known as the Kozak sequence [16]. Once the start codon is recognized, the 60S large ribosomal subunit joins to form a functional 80S ribosome, completing the initiation phase. During elongation, the ribosome moves along the mRNA in 5′ to 3′ direction, decoding the genetic information codon by codon. Each codon is matched by a complementary anticodon on an aminoacyl-tRNA (a tRNA charged with an amino acid) entering the aminoacyl (A)-site of the ribosome. The ribosome facilitates the formation of a peptide bond between the amino acid on the A-site tRNA and the peptide bound to the tRNA in the peptidyl (P)-site. This reaction is catalysed by the peptidyl transferase activity of the large ribosomal subunit. The ribosome translocates to the next codon, shifting the tRNA from the A-site to the P-site and freeing the A-site for the next aminoacyl-tRNA. Termination occurs when a stop codon (UAA, UAG or UGA) enters the A-site. Stop codons are recognized by release factors rather than tRNAs and release the newly synthesized protein. The ribosomal subunits, mRNA and tRNA subsequently dissociate, completing the translation process.

### 2.1. Initiation

**Figure 1.**
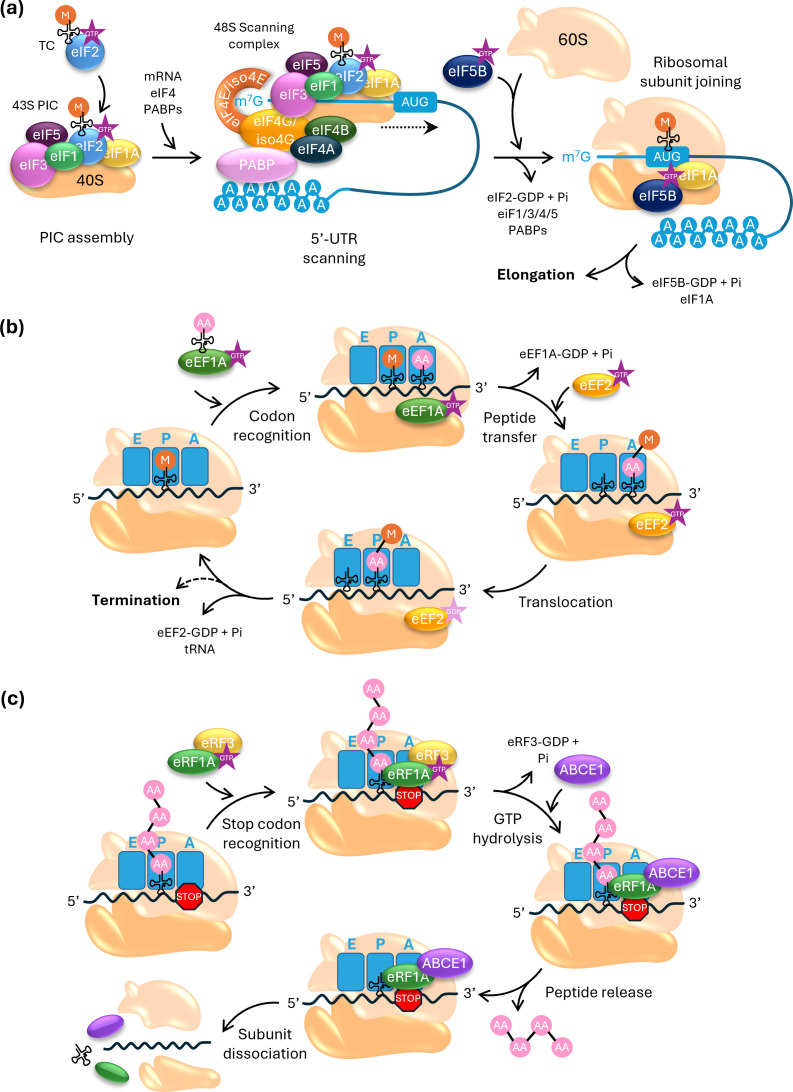
Overview of canonical translation in eukaryotes. (a) Translation initiation is governed by eukaryotic initiation factors (eIFs) and begins with the binding of the cap-binding complex (eIF4E and eIF4G) to the mRNA’s 5′ m^7^Gppp cap and the association of PABPs with eIF4G and the poly-A tail, resulting in mRNA circularization. eIF4G recruits DEAD-box helicases eIF4A and eIF4B to unwind RNA secondary structures. In parallel, the 43S pre-initiation complex (PIC), composed of the 40S ribosomal subunit, ternary complex (TC, consisting of eIF2-GTP and the initiator tRNA), eIF1, eIF1A, eIF3 and eIF5 assembles, binds to the mRNA and interacts with the cap-binding complex to form the 48S scanning complex. The 48S complex scans the 5′ UTR in 5′ to 3′ direction to identify the initiation codon, whereupon it adopts a closed conformation, stabilizing the initiator tRNA in the peptidyl (P)-site. eIF1 and eIF5 dissociate, triggering eIF2-GTP hydrolysis and the release of eIF2-GDP, eIF3, eIF4 and PABPs. Subsequently, eIF5B-GTP mediates 60S subunit joining with the aid of eIF1A. Hydrolysis of eIF5B-GTP results in the formation of the functional 80S ribosome, ready for peptide elongation. (b) The elongation cycle commences when an aminoacyl-tRNA, escorted by eukaryotic elongation factor 1A (eEF1A)-GTP, binds the mRNA codon in the ribosomal aminoacyl (A)-site, triggering GTP hydrolysis by eEF1A and securing the tRNA’s position. The ribosome’s peptidyl transferase centre (PTC) catalyses peptide bond formation between the A-site tRNA’s amino acid and the nascent polypeptide chain on the P-site tRNA, transferring the chain to the A-site. eEF2-GTP then binds, and GTP hydrolysis drives translocation of the ribosome along the mRNA, moving the peptidyl-tRNA to the P-site and the deacylated tRNA to the exit (E)-site. The A-site is freed for the next aminoacyl-tRNA, and the cycle continues until a stop codon signals termination. (c) Termination occurs when a stop codon (UAA, UAG or UGA) enters the ribosome’s A-site. Stop codons are recognized by eukaryotic release factor 1 (eRF1). eRF1, aided by eRF3, triggers hydrolysis of the bond between the polypeptide chain and the tRNA in the P-site, catalysed by the ribosome’s PTC. Following polypeptide release, ABCE1 facilitates the dissociation of ribosomal subunits, mRNA and deacylated tRNA.

In plants, as in other eukaryotic organisms, translation initiation is a meticulously orchestrated process (figure 1a). It involves various eukaryotic initiation factors (eIFs), the two ribosomal subunits, messenger RNA (mRNA), initiator transfer RNA (tRNA_i_), GTP, ATP and poly-A binding proteins (PABPs) and is regarded as the primary point of regulation [17]. Initiation begins with the binding of the cap-binding complex eIF4F to the mRNA’s 7-methyl guanosine (5′ m^7^Gppp) cap structure and of PABPs to the poly-A tail. The eIF4F complex comprises a cap-binding protein, eIF4E and a scaffolding protein, eIF4G, which also interacts with PAPBs, resulting in the circularization of the mRNA. eIF4G also recruits the DEAD-box helicases eIF4A and eIF4B to the mRNA to unwind its secondary structures.

The 43S PIC forms prior to interaction with the mRNA and contains the multi-factor complex (MFC) consisting of eIF1, eIF1A, eIF3 and eIF5, the 40S ribosomal subunit and the ternary complex (TC), which is composed of binding protein eIF2, GTP and Met-tRNA_i_. Then, the 43S PIC binds to the mRNA and its associated factors, eIF4F, PABP, eIF4A and eIF4B, to form the 48S scanning complex. This complex scans the 5′ untranslated region (5′ UTR) of the mRNA in a 5′ to 3′ direction to locate the initiation codon. This is usually an AUG, but the nucleotides situated immediately upstream and downstream strongly affect the efficiency, accuracy and specificity of translation initiation [16,18]. The initiation factors eIF1 and eIF1A play a crucial role in this process [19]. eIF3, the largest and most complex initiation factor, consists of 12 subunits and is essential for nearly all steps of translation initiation. It facilitates the loading of the TC on to the 40S ribosomal subunit, guides the 43S PIC to the mRNA cap by interacting with eIF4G and supports scanning and recognition of the initiation codon. Additionally, it prevents premature joining of the 40S and 60S ribosomal subunits until the initiation codon is reached and participates in the reinitiation process [17,20].

Once the start codon is identified, the 48S complex switches to a closed conformation, securing the Met-tRNA_i_ in the P-site and ejecting eIF1 and eIF5, which triggers the hydrolysis of the eIF2-bound GTP and the release of Pi and eIF2-GDP. Then, eIF5B-GTP is recruited, and together with eIF1A promotes the joining of the 60S subunit. Formation of the functional 80S initiation complex is accompanied by GTP hydrolysis on eIF5B, releasing eIF5B-GDP and eIF1A. The functional 80S ribosome can now proceed with peptide elongation [17,20].

### 2.2. Elongation

Elongation ensures the accurate and efficient extension of the polypeptide chain and involves three core steps: codon recognition, peptide bond formation and ribosome translocation (figure 1b). These steps occur cyclically as the ribosome moves along the mRNA. The process requires the coordinated action of eukaryotic elongation factors (eEFs), ribosomal subunits, aminoacyl-tRNAs and enzymatic components to ensure that amino acids are added in the correct sequence dictated by the mRNA codons.

The elongation cycle begins with the delivery of an aminoacyl-tRNA, escorted by eEF1A-GTP, to the ribosomal A-site. The tRNA’s anticodon pairs with the complementary codon on the mRNA, inducing a conformational change in the ribosome that stimulates GTP hydrolysis by eEF1A and positions the aminoacyl-tRNA securely in the A-site. Next, the ribosome’s peptidyl transferase centre, located in the 60S subunit, catalyses the formation of a peptide bond between the amino acid on the A-site tRNA and the nascent polypeptide chain attached to the P-site tRNA, thereby transferring the chain to the A-site tRNA. eEF2-GTP binds to the ribosome, and hydrolysis to eEF2-GDP facilitates a conformational shift that moves the ribosome’s three nucleotides in a 3′ direction along the mRNA. This shift relocates the peptidyl-tRNA from the A-site to the P-site and the deacylated tRNA from the P-site to the exit (E)-site, where it leaves the ribosome. Concurrently, the A-site becomes available to accommodate the next aminoacyl-tRNA. This cyclical process repeats until the ribosome encounters a stop codon, marking the transition to the termination phase [17].

### 2.3. Termination

Translation termination is the final stage of protein synthesis, where the nascent polypeptide chain is released from the ribosome (figure 1c). Termination begins when a stop codon (UAA, UAG or UGA) enters the A-site of the ribosome. Unlike sense codons, stop codons do not correspond to any tRNA. Instead, they are recognized by eukaryotic release factor 1 (eRF1). eRF1 has a highly conserved structure that allows it to specifically interact with the stop codon in the ribosome’s decoding centre, ensuring that translation halts precisely at the correct position. Once eRF1 recognizes the stop codon, it triggers the hydrolysis of the bond between the polypeptide chain and the tRNA in the P-site, catalysed by the ribosome’s peptidyl transferase centre. The process is facilitated by eRF3, a GTPase that interacts with eRF1. In plants, eRF3 enhances the termination efficiency by ensuring the correct positioning of eRF1 and providing energy through GTP hydrolysis [17,21]. Following eRF3-GDP release, ATP-binding cassette E (ABCE1)/RNase L inhibitor 1 (RLI1) is recruited by eRF1. ABCE1 induces a conformational change that releases the polypeptide and dissociates the ribosomal subunits, mRNA and deacylated tRNA to allow for subsequent rounds of translation [17,21].

### 2.4. Cap-independent translation

Eukaryotic mRNAs typically undergo canonical cap-dependent translation. However, under stress conditions such as heat shock, viral infection or nutrient deprivation, general translation initiation is often suppressed, prompting a shift to cap-independent translation, a mechanism that enables protein synthesis without the need for the 5′ cap structure. Instead, it relies on specific RNA sequences or structures within the mRNA to recruit the ribosome for protein synthesis [22].

The most extensively studied mechanism of cap-independent translation involves internal ribosome entry sites (IRESs). IRESs are highly structured RNA elements situated in the 5′ UTR of some mRNAs. They directly recruit the ribosome along with associated initiation factors to initiate translation. IRESs were first identified in viral RNAs, such as those of poliovirus, which lack a 5′ cap and rely entirely on IRES-mediated translation [23,24]. Cellular IRESs can be broadly categorized into two types based on their ribosome recruitment mechanisms: (i) type I IRESs facilitate ribosome interaction through the binding of IRES-transacting factors to specific *cis* elements, and (ii) type II IRESs utilize a short *cis* element that directly pairs with the 18S ribosomal RNA (rRNA) for ribosome recruitment [22].

Beyond IRESs, other cap-independent translation mechanisms have been identified, including those involving RNA modifications like N6-methyladenosine (m⁶A) [25]. These modifications can recruit specialized reader proteins, such as eIF3, which mediate ribosome recruitment and translation initiation.

### 2.5. Plant-specific components of translation

Although plants share fundamental characteristics of the mRNA translation machinery with other eukaryotes, they have developed unique features. In addition to the canonical eIF4F complex, plants harbour a specialized eIFiso4F complex, composed of eIFiso4E and eIFiso4G. Plant eIF4E and eIFiso4E share 50% amino acid sequence similarity and have comparable molecular weight. Similarly, eIFiso4G and eIF4G in plants are alike in their C-terminal regions, which include the eIF4E binding site and two HEAT domains; however, eIFiso4G lacks the N-terminal region present in eIF4G [26].

eIF4F and eIFiso4F have redundant but also distinct functions. *In vitro*, the translation activity of mixed eIF4E–eIF4isoG or eIFiso4E–eIF4G complexes is primarily determined by the eIF4G or eIFiso4G subunit. Different mRNA templates displayed varying responses to eIF4F or eIFiso4F, suggesting that these complexes selectively discriminate between mRNAs [27]. While eIFiso4F primarily supports the translation of standard mRNAs, eIF4F appears to be more adept at facilitating the translation of non-standard mRNAs, such as uncapped mRNAs, those with structured 5′ UTRs or dicistronic mRNAs. It also functions effectively under conditions where cap-dependent translation is inhibited [28].

## 3. Translation factors under heat stress

**Figure 2.**
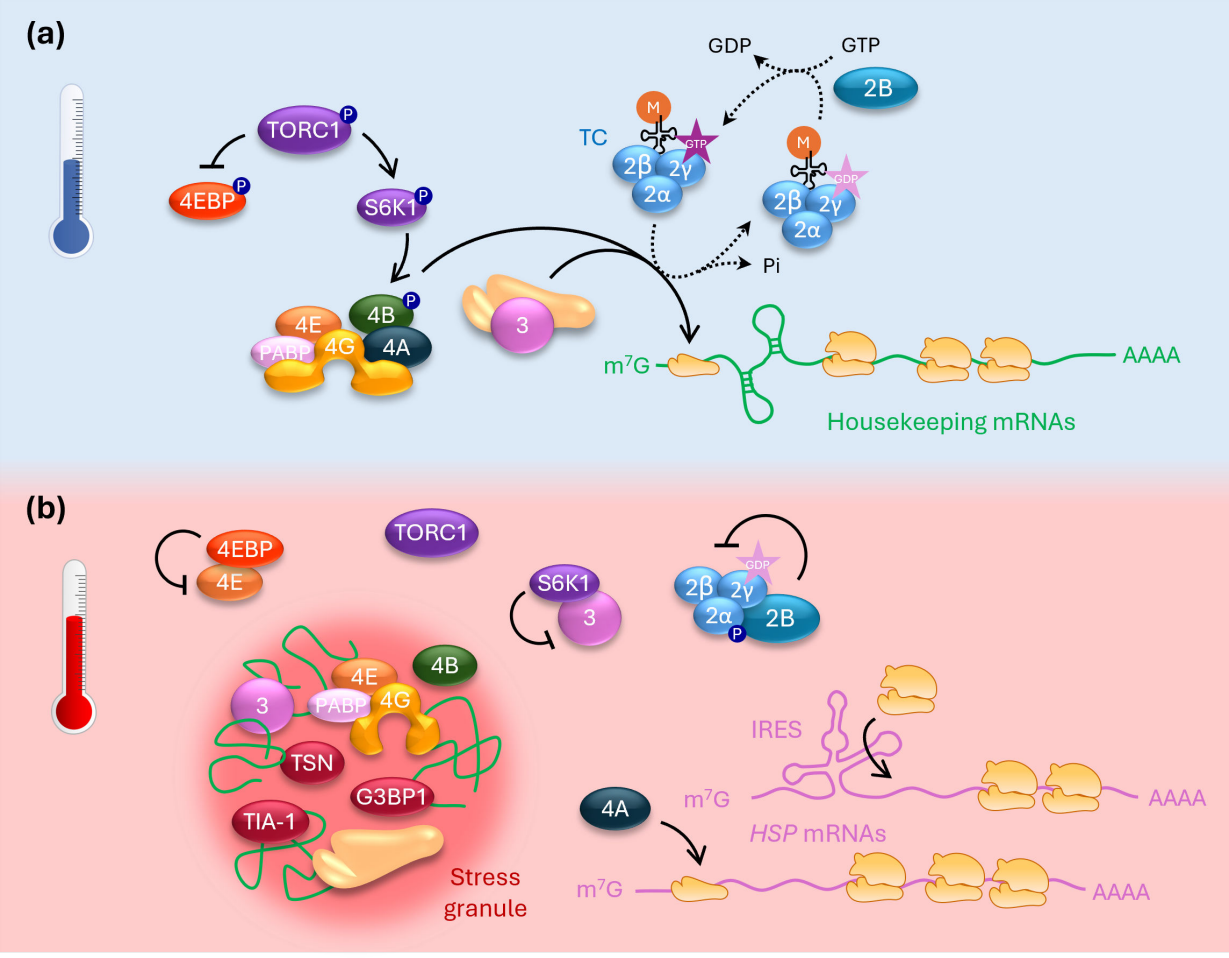
Regulation of eukaryotic initiation factors during heat stress. (a) Under non-stress conditions, eukaryotic initiation factors promote canonical translation initiation, resulting in efficient translation even of structurally complex housekeeping mRNAs. Active TOR complex 1 (TORC1) promotes translation via phosphorylation of eIF4E binding protein (4EBP) and ribosomal protein S6 kinase 1 (S6K1), inhibiting 4EBP but activating S6K1. S6K1 subsequently phosphorylates eIF4B, leading to its recruitment by eIF4A/eIF4G. In parallel, active, GTP-containing ternary complexes (TCs) are constantly replenished by the GDP–GTP exchange factor eIF2B. (b) Under heat stress, hypo-phosphorylated 4EBP binds to and inhibits eIF4E, while inactive S6K1 does the same with eIF3. Additionally, the TC’s eIF2α subunit becomes phosphorylated, causing the TC to be sequestered by eIF2B. eIF4G undergoes a conformational change that leads to the release of eIF4A. The eIF4E/4G complex, alongside other initiation factors, the 40S ribosomal subunit and many housekeeping mRNAs, is recruited into stress granules. These processes jointly cause a global reduction in canonical translation. eIF4A can independently promote the translation of some structurally simple mRNAs encoding heat shock proteins (HSPs), while other heat-induced mRNAs employ an internal ribosomal entry site (IRES) to enable cap-independent translation. Numbers represent initiation factors, solid arrows indicate positive regulation and perpendicular lines indicate inhibition. The processes depicted here were shown to regulate translation under heat stress in animals and/or yeast; while many signalling components are conserved, it is currently unclear whether the same mechanisms operate in plants.

Given that initiation is the rate-limiting step of the translation process, initiation factors play crucial roles in tailoring translation rates to both intrinsic and external stimuli, including temperature. A recent study in yeast found the cap-binding complex eIF4F to have a major role in responding to heat shock. Under control conditions, eIF4F interacts with eIF4A, eIF4B and PABP [17]. However, heat stress causes structural reorganization of the eIF4A-binding domain of eIF4G, leading to the dissociation of eIF4A and the formation of arrested heat shock mRNA ribonucleoprotein (HS-mRNP) complexes containing eIF4E, eIF4G and many housekeeping mRNAs. eIF4A independently promotes the translation of heat shock mRNAs [29], possibly relying on their short and simple 5′ UTRs, which allow eIF4A to function without eIF4G (figure 2) [30].

In animal cells, heat shock causes a global reduction in protein synthesis, which involves phosphorylation of the *α*-subunit of initiation factor eIF2. eIF2 delivers the initiator tRNA to the ribosome, upon which its bound GTP is hydrolysed to GDP. eIF2-GDP must then be regenerated to eIF2-GTP to restore its activity, which requires the guanine nucleotide exchange factor eIF2B. Phosphorylation of eIF2α on residue S52 in yeast increases affinity to eIF2B, causing eIF2 to be sequestered by eIF2B and preventing its regeneration (figure 2) [31,32]. In mammals, phosphorylation of eIF2α is mediated by several stress-responsive kinases, with heme-regulated inhibitor (HRI) being the major kinase promoting eIF2α phosphorylation upon heat shock [33]. Heat-induced eIF2α phosphorylation also occurs in yeast but is not required for global downregulation of translation [34].

In plants, eIF2α phosphorylation increases in response to stresses such as wounding, infection, cold, high light, UV radiation and herbicide treatment, mediated by the kinase general control non-derepressible 2 (GCN2) [35–37]. Its importance for overall protein synthesis, however, remains debated as eIF2α phosphorylation does not always correlate with global translation rates [38]. Phosphorylation of other plant initiation factors may also play a role in environmental responses. Phosphorylation of eIF3B, eIF4A, eIF4B, eIF4G and eIFiso4G was found to change in response to environmental factors such as light, heat, CO_2_ and hypoxia [39–41]. In wheat, heat shock primarily affects eIF4A and eIF4B, which undergo increased phosphorylation and dephosphorylation, respectively, while minimal changes are observed for eIF4E and eIF2α [39]. The functional relevance of these phosphorylation changes remains unknown.

For some initiation factor mutants, heat-dependent phenotypes have been described. In rice, *eif3h* knockout mutants exhibit pronounced developmental defects, such as reduced plant height, fewer tillers and decreased fertility under high-temperature conditions [42]. Together with adaptation to environmental temperature 1 (AET1) and receptor for activated C kinase 1 (RACK1), eIF3h enhances the translation efficiency of transcripts encoding auxin response factor 19 (OsARF19) and OsARF23, which are key regulators of auxin signalling and ensure normal development under high temperature [42].

In the model plant *Arabidopsis thaliana* (Arabidopsis), the *hot3-1* allele, which contains a single point mutation in the *eIF5B1* gene, delays recovery of polysome loading after heat stress and reduces translational efficiency of a subset of stress-protective transcripts. These defects correlate with impaired heat acclimatization of the *hot3-1* mutant [43]. The study proposed that reduced levels of eIF5B1 during the recovery phase after heat treatment cause an imbalance in factors needed to reinitiate translation. Additionally, the decreased translation efficiency of many newly transcribed stress-tolerance genes probably exacerbates the heat sensitivity observed in *hot3-1* plants.

Evidence for a specific role of translation elongation and termination factors involved in heat shock responses remains scarce. Transgenic lines overexpressing constitutively GTP- and GDP-bound eEF1A variants exhibit markedly reduced tolerance to heat stress at both seedling and mature stages, indicating that dynamic GTP-GDP cycling of eEF1A is critical for heat stress regulation. In contrast, the homozygous T-DNA knockout mutants of *eEF1A* do not display a heat-sensitive phenotype [44]. A role in enhancing stress tolerance has also been reported for eIF5A, a protein initially identified as an initiation factor but later found to participate in elongation [45]: overexpression of *Rosa chinensis* eIF5A in Arabidopsis significantly improves the plants’ ability to withstand thermal stress [46]. Clearly, further research is required to fully appreciate the importance of translation elongation and termination factors under high-temperature conditions.

## 4. Heat-induced biomolecular condensates and their role in translation

Biomolecular condensates, which form so-called membrane-less organelles within the cell, have attracted substantial attention over the last decade. These structures represent a cellular sorting mechanism that spatially organizes and concentrates specific biomolecules without the need for membrane boundaries and transport systems. Recent evidence suggests that the formation of these condensates is driven, or at least facilitated, by liquid–liquid phase separation (LLPS). LLPS leads to the segregation of molecules into a dense and a dilute phase, a process influenced by factors such as pH, temperature and salinity, as well as the concentration and intrinsic properties of the biomolecules themselves, including the presence of polyvalent or unstructured domains [47,48].

In eukaryotic cells, processing bodies (P-bodies) and stress granules (SGs) represent two important groups of cytoplasmic ribonucleoprotein condensates [49,50]. P-bodies contain components of the RNA degradation machinery as well as untranslated mRNAs; they are present in non-stressed cells, although their formation is further induced under stress. SGs, on the other hand, form rapidly in response to stress and, alongside mRNAs, contain many translation initiation factors as well as the 40S ribosomal subunit (figure 2b), suggesting that the enclosed mRNAs are stalled in the process of translation initiation [49,51]. Global downregulation of translation under heat shock is, however, not dependent on SG formation, suggesting that heat-induced SGs (HSGs) are a consequence of translation inhibition rather than its cause [52–54]. Below, we briefly describe the dynamics of SGs in the heat response; for a more detailed discussion of SG composition and function, readers are referred to some excellent recent reviews [47,48,51,55].

Formation of SGs relies on scaffold proteins such as the Ras GTPase-activating protein-binding protein 1 (G3BP1), T-cell-restricted intracellular antigen-1 (TIA-1) and Tudor staphylococcal nuclease (TSN) in mammals [54,56,57] as well as the TIA-1 homologue RNA-binding protein 47 (RBP47) and TSN in plants [58–60]. A recent study identified the disordered protein Fusang Tree 1 (FUST1) as a bona fide plant thermosensor that condenses in response to rising temperature and is involved in the early stages of HSG assembly [61]. Alongside these core components, translation initiation factors such as eIF3, eIF4B, eIF4E, eIF4G and PABPs, as well as elongation factor eEF1B, constitute prominent constituents of SGs [29,62–66]. eIF4G takes on a key role in promoting HSG assembly after heat-induced structural rearrangement [29]. In contrast, eIF2 is notably absent from HSGs, which led to the hypothesis that low availability of active TCs contributes to HSG formation [67]; in mammals, but not yeast, this may depend on eIF2α phosphorylation [34,68,69].

HSGs are generally associated with thermotolerance. While not strictly necessary for global translational shutdown under heat stress, they have been implicated in reduced translation of specific transcripts. Heat-induced condensate formation of the yeast DEAD-box RNA helicase Ded1p, which acts alongside eIF4A, has been functionally linked to reduced translation of structurally complex housekeeping mRNAs while simultaneously promoting the translation of structurally simpler mRNAs encoding HSPs [70]. This aligns with findings that *HSP70* and *HSP90* mRNAs are excluded from HSGs [71]. However, not all mRNAs sequestered in SGs are translationally repressed: using single-molecule imaging, Mateju *et al.* [72] showed that mRNAs can complete the entire translation cycle in SGs, which is consistent with the presence of elongation and termination factors in some HSGs [34,66].

It has been proposed that SGs serve as reservoirs for mRNAs and initiation complexes, facilitating the coordinated resumption of translation during stress recovery [49]. Indeed, SGs disassemble over a period of 1−4 h [63], which roughly correlates with restored protein synthesis [69]. Disassembly requires the chaperon function of HSPs, including HSP70, HSP104 and small HSPs, which are upregulated under heat stress and subsequently recruited to HSGs during recovery [63,73–75]. Interestingly, HSPs seem to be integral components of plant SGs that form under long-term heat exposure. In contrast to generic SGs that appear rapidly after heat exposure, Weber *et al.* [76] found these plant-specific HSGs to consist primarily of HSPs and to lack RBP47, UBP1 and, notably, mRNAs. However, this distinction has been questioned, as small HSPs were found to be part of condensates containing translation factors and mRNAs [74].

Some observations suggest that HSGs also contribute to a plant’s thermal memory, the process by which a past heat exposure modifies the response to subsequent stress events, resulting in so-called acquired thermotolerance. Transcripts encoding HSFA2, an essential component of thermal memory [77], localize to HSGs [78], and *fust1* mutants are impaired in acquired thermotolerance [61]. Whether mutations in other HSG components similarly affect acquired thermotolerance remains unclear, and further research is needed to clarify the role of HSGs in thermal memory.

## 5. Translational control by the target of rapamycin pathway

The target of rapamycin (TOR) kinase represents a key sensor of the cell’s energy status in eukaryotic organisms. In yeast and mammals, TOR complex 1 (TORC1) becomes inactivated under low energy levels and various stresses, resulting in a global downregulation of protein synthesis [79]. This downregulation is mediated by reduced phosphorylation of several TORC1 substrates: (i) hypo-phosphorylated eIF4E-binding protein (4EBP) binds to eIF4E, inhibiting eIF4F assembly [80–82]. (ii) Active ribosomal protein S6 kinase 1 (S6K1) phosphorylates eIF4B, promoting its recruitment into the PIC, while inactive, hypo-phosphorylated S6K1 interacts with eIF3, thereby interfering with translation initiation (figure 2) [83]. Additionally, S6K1 regulates eEF2 kinase (eEF2K) and thereby reduces eEF2 activity, downregulating elongation [84,85]. (iii) Hypo-phosphorylated La-related protein 1 (LARP1) acts as a translational repressor for mRNAs containing a 5′-terminal oligopyrimidine (TOP) motif, which in mammals includes many components of the translation machinery [86–89]. Heat shock reduces TORC1 activity in mammalian epithelial cells [90] and triggers TORC1 recruitment into SGs in yeast [91], but TORC1’s precise contribution to the global heat-induced shutdown of translation is still debated.

In plants, several of the above-mentioned TOR signalling pathways appear to be conserved, including 4EBPs [92], S6K1/eIF3 [93] and LARP1/5′-TOP mRNAs [94]. Additionally, the TOR antagonist Snf1-related kinase 1 (SnRK1) assumes a direct role in translational regulation, phosphorylating eIF4E and eIFiso4E, reducing translation initiation [95]. TOR contributes to plant thermotolerance [96], but it remains unclear whether any of these pathways are specifically activated or modulated during heat stress.

## 6. Translational regulation via RNA secondary structures

RNA folding is inherently temperature-dependent; thus, RNA secondary structures are intrinsically thermosensitive and well suited for the perception of temperature signals. In bacteria, so-called RNA thermometers effectively tailor translation to the temperature environment: these secondary structures form across the ribosome binding site, interfering with translation initiation at low temperatures; as temperatures rise, these structures melt or rearrange, exposing the ribosome binding site and allowing for initiation to occur [13].

While prevalent in bacterial systems, few thermosensitive RNA structures with regulatory functions have been described in eukaryotes. In the parasite *Leishmania*, a polypyrimidine tract (PPT) located in the 3′ UTR of *Hsp83* mRNA enhances translation at elevated temperatures. The PPT undergoes partial melting during a shift from 26°C to 37°C, and it is proposed that the more open structure facilitates interaction with factors at the 5′ end during mRNA circularization, promoting translation [97]. In Arabidopsis, a thermosensitive hairpin structure resides in the mRNA encoding the transcription factor phytochrome interacting factor 7 (PIF7), which plays a pivotal role in morphological acclimatization to higher temperatures. Upon a shift from 17°C to 27°C, this hairpin structure adopts a more relaxed conformation that enhances translation [10]. A similar structure is found in the Arabidopsis *HSFA2* transcript [10], which encodes a key factor for thermal memory, but any translational regulation is probably secondary to the strong transcriptional induction and alternative splicing of *HSFA2* under heat stress [77,98]. In other examples, the involvement of structural rearrangements is even less clear: the Drosophila *Hsp90* mRNA displays extensive structure formation in its 5′ UTR that may restrict its translation at lower temperatures, while AUG-proximal nucleotides are specifically required for *Hsp90*’s efficient translation upon heat shock [99]. It is unknown whether structural changes contribute to this process.

Structural elements in 5′ UTRs also play a critical role in cap-independent translation under heat stress. Human *Hsp70*, mammalian *immunoglobulin binding protein* (*BiP*) and maize *HSP101* mRNAs all harbour IRESs in their 5′ UTRs [100–102]. While these elements themselves may not have a thermosensory function, they are essential for efficient translation under heat stress when eIF4F function is impaired (figure 2b).

## 7. Regulatory functions of upstream open reading frames under heat stress

Upstream open reading frames (uORFs) are common 5′ UTR features of eukaryotic transcripts—found in approximately 50% of human mRNAs and 40% of Arabidopsis mRNAs [103]. The function of uORFs is complex and not fully understood, but they are thought to directly impact translation efficiency by consuming PICs and stalling ribosomes [104]. Newly formed uORFs are generally considered to be deleterious and undergo purifying selection, although a subset has evolved to regulate translation [105]. Recently, uORFs have emerged as modulators of temperature-dependent stress responses in eukaryotes, including plants.

Temperature-dependent translation of uORFs has been observed in yeast, with most uORFs displaying increased translation rates at higher temperatures [106]. In several genes that mainly contain uORFs with a canonical start codon, this leads to reduced translation of the main ORF. However, another study of stress-dependent uORF translation in yeast indicates that increased uORF translation is largely unrelated to downstream translation but that a small subset of uORFs exists that exhibit reduced translation under stress, correlating with elevated downstream translation rates [107].

In the bread mould *Neurospora*, temperature-sensitive accumulation of the circadian clock protein frequency (FRQ) is controlled by a dual mechanism: temperature-dependent splicing of the translational start site and translational inhibition by multiple uORFs at low temperatures [108]. The uORFs’ temperature dependency has been linked to their non-canonical start codons, which are preferentially initiated at lower temperatures.

uORFs have been implicated in the integrated stress response in animals and yeast. Under stress conditions, phosphorylation of eIF2α reduces initiation at canonical start codons, an effect which is escaped by some stress-related mRNAs [109]. A well-characterized example is the mRNA encoding vertebrate activating transcription factor 4 (ATF4), which contains two uORFs in its 5′ UTR: a short uORF1 that promotes and a longer uORF2 that overlaps out of frame with, and hence interferes with translation of, the main ORF [110]. Under non-stress conditions, ribosomes rapidly acquire a new TC and efficiently reinitiate at uORF2 after translation of uORF1, repressing the main ORF's translation. Under stress, eIF2α phosphorylation reduces the availability of TCs, thereby suppresses reinitiation at uORF2 and favours reinitiation at the main ORF. A similar mechanism involving four uORFs operates for the yeast ATF4-homologue GCN4 [111], but its parallels in plants remain unclear. While mammals possess four eIF2α kinases, plants possess only one—GCN2. A direct role for plant GCN2 in translational regulation under stress has yet to be demonstrated, despite evidence that both GCN2 and eIF2α phosphorylation are modulated under various stress conditions [35–37].

eIF2A is another initiation factor linked to uORF-dependent translational regulation. In human T cells, it is required for maintaining *BiP* translation during the integrated stress response, and this regulation is imparted by two non-canonical start codon uORFs in the 5′ UTR [112]. In this case, persistent uORF translation mediated by eIF2A shelters the *BiP* mRNA from translational shutdown under stress; the exact mechanism, however, remains unknown.

Although uORFs are rarely conserved at the sequence level across species, conserved peptide uORFs (CPuORFs) exist in eukaryotic genomes and serve regulatory functions [113]. In Arabidopsis*,* CPuORFs have been identified as regulating the translation of their downstream ORFs in response to stress [114]. For example, CPuORF46 causes ribosomal stalling under control conditions but promotes a substantial increase in downstream reporter gene activity in response to heat [114]. Its conservation across flowering plants and ferns suggests that it is functionally relevant, though the precise heat-dependent mechanism remains uncharacterized. Another CPuORF represses the translation of the Arabidopsis *heat shock factor B1* (*HSFB1*). Upon heat shock, this repression is lifted, allowing HSF1B protein to accumulate and promote the expression of HSPs [115]. As with CPuORF46, the CPuORF’s effect is reproducible on reporter genes, highlighting that uORFs can function as independent temperature-sensitive regulators. However, how CPuORFs themselves are regulated by temperature remains an open question.

## 8. Temperature-regulated RNA modifications

RNA modifications provide a rapid and reversible means for regulating the fate of transcripts in response to environmental changes. They are diverse—more than 170 RNA modifications have been identified to date [116]. Of these, the m^6^A modification is the most abundant and well-characterized modification [117], with known roles in translation regulation, particularly through modification of the 5′ UTR of mRNAs [25].

In mammalian cells, heat shock induces a rapid shift of the m^6^A reader YTHDF2, a protein required to decode m^6^A signals, into the nucleus [118]. Although YTHDF2 does not have the capacity to write m^6^A itself, YTHDF2 relocation results in the accumulation of m^6^A modifications largely in the 5′ UTR across the transcriptome. These modifications are associated with elevated cap-independent translation of stress-induced transcripts, including those encoding HSPs. A single m^6^A mark can bind to eIF3 and thereby recruit the PIC in the absence of the cap-binding component eIF4E [25,118].

In Arabidopsis*,* studies on m^6^A dynamics during heat stress yielded contrasting results: while one study observed only minor effects on global m^6^A levels [119], another identified heat stress as a major driver of differential methylation events compared with other abiotic conditions [120]. Prolonged cold stress, on the other hand, results in an overall reduction in m^6^A modifications in 3′ UTRs, and disruption of m^6^A methylation strongly reduces cold tolerance [121,122]. Notably, translational regulation of approximately one-third of the Arabidopsis transcriptome is disrupted in the absence of m^6^A, and transcripts enriched with cold-induced m^6^A marks exhibit increased ribosome occupancy [121]. Whether heat-induced m^6^A modification dynamics also lead to altered ribosomal occupancy or transcript stability remains to be experimentally determined.

While the effects of m^6^A on translation vary depending on species and context, 7-methylguanosine (m^7^G) at the 5′ cap structure has a universal role in promoting canonical translation initiation [123]. It has traditionally been considered a constitutive property of mRNAs, and its dynamics are only beginning to be explored [124]. Internal m^7^G modifications in mRNA, on the other hand, increase dramatically following heat shock, which directly impacts translation efficiency in mammalian cells [125]. Additionally, transcripts bearing internal m^7^G modifications are shuttled to SGs to attenuate translation [126]. Whether these effects are conserved in plants is currently unknown.

mRNAs are not the only type of RNA affected by modifications. In *Caenorhabditis elegans*, 5-methylcytidine (m^5^C) modifications are reliably detected in tRNAs and rRNAs; loss of m^5^C results in translational stalling at UUG‐rich transcripts and impairs fertility at elevated temperatures [127]. In rice, the tRNA^His^ guanylyltransferase AET1 is required for normal growth under heat stress and controls the translation efficiency of multiple *ARF* mRNAs [42]. Additionally, naturally occurring variants of the rice tRNA 2-thiolation gene *Slender Guy 1* (*SLG1*) confer thermotolerance by increasing thiolated tRNA levels [128]. In yeast, aberrant tRNA thiolation leads to impaired translation rates [129], and components of the ubiquitin-related modifier 1 (URM1) pathway, which regulates thiolation of tK^UUU^, tQ^UUG^ and tE^UUC^ tRNAs, are downregulated at elevated temperatures, resulting in altered translation rates for transcripts biased in those codons [130].

Similarly to tRNA modifications, selective rRNA modifications can alter the translation efficiency of some codon-biased genes [131]. The hyperthermophile archaeon *Thermococcus kodakarensis* contains 5-methylcytidine (m^5^C) modifications at rRNA sites critical for translation, and a reduction in m^5^C modification strongly impairs growth at high temperatures of 85°C and above [132]. Whether this is a necessity for extremophile survival and whether rRNA modifications are consequential to eukaryotic temperature responses remains to be determined. Recent findings that plant rRNA modifications such as m^6^A and pseudouridylation modulate translation and cold tolerance [133,134] raise the possibility that rRNA modifications could also be relevant for the plant’s heat response.

RNA modifications provide a fine-tuning mechanism for protein synthesis, and natural variations in these modifications are emerging as potent regulators of abiotic stress resilience [135]. Existing methods for detecting RNA modifications have unique drawbacks in their accuracy, sensitivity and throughput and are often disrupted by secondary structures that are common to tRNAs and rRNAs [136]. Methods that permit the routine and accurate detection of RNA modifications—particularly at the individual transcript resolution—will be necessary to improve the accessibility of identifying and quantifying their dynamics in temperature. Recently, nanopore direct RNA sequencing has been demonstrated to be a promising tool to detect m^6^A modifications at single nucleotide resolution, and where high-quality training datasets are available, it shows promise in the detection of rare modifications [137]. Other approaches, such as Cas13-targeted RNA modifications, will be helpful in resolving the role of specific transcript modifications in translation-driven temperature responses [138]. A recent demonstration of the efficacy of Cas13-driven m^6^A modifications in plants marks its potential for improving translational regulation during temperature responses [139].

## 9. Conclusion

Translational regulation has been studied in eukaryotic systems for decades, but many of its intricate mechanisms have only begun to be unravelled in plants. Establishing techniques such as ribosome profiling, initiation profiling and translating ribosome affinity purification sequencing in plants has substantially advanced our ability to map global changes in translation rates [140], while many methods for tracking single translation events in real time [141–143] are yet to be adapted for plant systems. As the toolkit for studying translational processes continues to expand, our understanding of plant translational processes—and how they are regulated in accordance with the temperature environment—will grow, offering new avenues for tuning plant translation and improving crop performance.

Current efforts to enhance plant performance often rely on transcriptional regulation, but translational mechanisms have the advantage of directly targeting existing transcripts, enabling rapid and dynamic responses to environmental stimuli. This is particularly relevant under fluctuating or acute stress conditions, when rapid cellular reprogramming may be critical for fitness and survival. However, the use of translational mechanisms to improve heat tolerance in crops remains scarce. Overexpression of the SG-associated protein TaMBF1c has been used to boost thermotolerance in wheat by enhancing the translation of heat stress-responsive transcripts [144], but such changes are constitutive and may lead to unintended consequences.

The addition or modification of *cis*-regulatory elements in UTRs allows for a more precise intervention in a transcript-specific manner. Advances in CRISPR/Cas-based gene editing have made the *in situ* modification of *cis* elements such as uORFs and RNA secondary structures feasible [145]. Indeed, this approach has already shown promise: modifying uORF sequences has improved disease resistance in rice [146], vitamin C content in lettuce [147] and sugar content in strawberries [148]. Similar strategies could be employed to improve heat resilience by enhancing the translation of protective proteins, such as molecular chaperones or reactive oxygen scavengers, under high temperature. Integrating such translational mechanisms into crop improvement strategies may unlock new possibilities for generating plants that can thrive under increasingly challenging temperature conditions.

## Data Availability

This article has no additional data.
